# Endoplasmic Reticulum Stress Promotes Prostate Cancer Cells to Release Exosome and Up-regulate PD-L1 Expression via PI3K/Akt Signaling Pathway in Macrophages

**DOI:** 10.7150/jca.81933

**Published:** 2023-04-17

**Authors:** Wei Xu, Meiyi Lu, Siqi Xie, Dangui Zhou, Mei Zhu, Chaozhao Liang

**Affiliations:** 1 Department of Urology, The First Affiliated Hospital of Anhui Medical University, Hefei, Anhui, PR China; 2 Institute of Urology, Anhui Medical University, Hefei, Anhui, PR China; 3 Department of Blood Transfusion, The First Affiliated Hospital of Anhui Medical University, Hefei, Anhui, PR China; 4 Department of Clinical Laboratory, The Affiliated Chaohu Hospital of Anhui Medical University, ChaoHu, Anhui, PR China

**Keywords:** Exosomes, Endoplasmic reticulum stressed, Prostate cancer cells, PI3K/Akt, Macrophages

## Abstract

Mounting evidence has demonstrated that endoplasmic reticulum stress (ERS) serves an important role in shaping the immunosuppressive microenvironment by modulating resident tumor-associated macrophages (TAMs). However, the communication between ER‑stressed tumor cells and TAMs is not fully understood. Exosomes have been reported to play a vital role in intercellular communication. Therefore, in order to investigate the role of ER stress‑related exosomes in prostate cancer cells promoting macrophage infiltration and polarization, laser scanning confocal microscope, RT-PCR, flow cytometric analysis, western‑blotting and cytokine bead array analyses were performed.The results demonstrated that TG-EXO downregulated the expression of PD-L1 on macrophages through flow cytometry analysis. In addition, Compared with CON-EXO, the expression of macrophage-associated inflammatory cytokines IL-12, TNF-α and IL-1βwas significantly decreased in TG-EXO treatment (*P*< 0.05). TG-EXO upregulated the expression levels of IL-6, IL-10 and TGF-β cytokinesin macrophages. Our research shows that TG-EXO increased PI3K/AKT signaling pathway compared to the CON-EXO group. In summary, we found exosomes from TG-treated prostate cancer cells altered the immunosupression status and affected macrophages polarization by up-regulating the expression of PD-L1 and inflammatory factors and PI3K/AKT pathway.

## Introduction

Prostate cancer (PCa) is a malignant tumor that seriously threatens men's health. In recent years, the incidence and mortality of PCa are increasing. The prostate specific antigen (PSA) detection has been questioned for its specificity and sensitivity, and prostate cancer cases have been often found to be advanced and lost the best period of surgical treatment 1. Despite considerable advances in treatment, the high incidence of secondary metastases still leads to a poor prognosis 2. Therefore, it is urgent for clinical and basic research to explore the underlying mechanism of PCa and find effective treatment methods.

Endoplasmic reticulum stress (ERS) is a necessary step for misfolded or unfolded proteins to return to their correct conformation and to further process under pathological conditions such as Ca^2+^ disorder, oxidative stress, viral, bacterial infection and cell hypoxia. It is a mechanism of cell self-protection. There is a very complex relationship between ERS and the body's immune system. Tumor cells in ERS state can suppress the immune system through a variety of mechanisms, thus lead to immune escape 3.

Tumor and surrounding stromal cells constitute the tumor microenvironment (TME), which provides an opportunity for the interaction among cancer, fibroblasts, inflammatory cells and microcapillaries. TME contains tumor-induced cytokines, growth factors and a variety of immune cells (including macrophages), which plays an important role in immunosuppression. Macrophages have high functional plasticity and heterogeneity. Phenotype and functional transfer of macrophages in TME often affect the occurrence and development of diseases. Tumor-associated macrophages (TAMs) are abundant in prostate tumors. TAMs can be either cytotoxic M1 macrophages or tumorigenicity M2 macrophages 2. M2 macrophages are involved in wound repair, angiogenesis, immunosuppression, and tumor enhancement. M2 macrophages also promote Thelper 2 (Th2) immune response 4. M1 macrophages are characterized by releasing cytotoxic free radicals and inhibiting tumor cells, promoting T-helpercell type 1(Th1) response 5. Abnormal metabolic factors can also aggravate the phenotype of these cells, such as hypoxia and ischemia. Low pH TME can induce ERS in tumor cells, thereby enhancing the immunosuppressive ability of TAMs and promoting macrophage infiltration and polarization. Recent studies have shown that tumor cells can transmit ERS signal to relevant immune cells in the tumor microenvironment, such as macrophages, resulting in changes in ERS-related gene transcription and coding, accompanied by increased secretion of pro-inflammatory cytokines 6.

Exosomes are 30-120 nm in diameter extracellular vesicles with a double-membrane structure that carry a variety of biological functions, such as miRNAs, mRNAs, lncRNA proteins, lipids and virus particles 7. Exosomes are released by exocytosis of vesicles, and substances in vesicles can be transferred and alter the signaling pathways of recipient cells. Exosomes are largely involved in tumor growth and progression, but they can also play an anti-tumor role depending on the cell type and microenvironment, for example, when cancer cells secrete more exosomes in response to stress conditions, such as hypoxia. Therefore, it is of great clinical significance to further study how tumor cells transmit ERS signals to macrophages in the tumor microenvironment, so as to change their polarization function.

Current studies generally believe that ERS is closely related to tumor immunosuppression, but there are still a lot of gaps to clarify how tumor cells secrete exosomes in the state of ERS, act on immune cells in their surrounding tumor microenvironment (especially change the polarization of macrophages), and then cause tumor immune escape. Therefore, the purpose of this study is to explore how tumor cells in the tumor microenvironment transmit ERS signals to macrophages through exosomes, up regulate the expression of programmed death receptor ligand 1 (PD-L1) through PI3K/AKT signaling pathway, then change its polarization properties, and result in tumor immune escape.

## Materials and methods

### Cell culture

The human prostate cancer cell line PC3 and human monocytic leukemia cell line THP-1 were purchased from Shanghai cell bank of Chinese Academy of Sciences. PC3 cells were cultured in high-glucose DMEM, 10% fetal bovine serum (FBS), 100 U/ml penicillin, and 100 mg/ml streptomycin. THP-1 cells were cultured in high-glucose RPMI-1640, 10% fetal bovine serum (FBS), 100 U/ml penicillin, and 100 mg/ml streptomycin. PC3 and THP-1 cells were incubated at 37 °C in a humidified 5% CO_2_ atmosphere. THP-1 monocytes were differentiated into macrophages with 100 ng phorbol 12-myristate 13-acetate (PMA, Sigma, P8139) within 48 h.

### Exosome Isolation and Characterization

We firstly collected the cell supernatants, and centrifuged at 3000 g for 15 min to remove cell debris. Then 2 ml ExoQuick precipitation solution and 10 ml cell supernatant was added to the supernatants 10 ml, and the mixture was refrigerated overnight (at least 12 h) at 4 °C. Next, the mixture was centrifuged at 1500 g for 30 min and the supernatants were sucked out. The remaining ExoQuick-TC solution was spun down by centrifugation at 1500 g for 5 min. Finally we resuspended the sediment with 160 µl PBS and subpackaged before being stored at -80°C. BCA protein assay kit (Beyotime Institute of Biotechnology, China) was used to quantify and standardize the protein concentrations in exosomes. The exosomes are designated as untreated cultured PC3 cells (CON-EXO) or PC3 cells treated with TG (TG-EXO).

### 2.3 Transmission electron microscope (TEM)

The purifified exosome pellets were diluted in sterile PBS and 10 µl suspensions were placed on copper grids for 2 min at room temperature. Then exosomes were stained with 2% phosphotungstic acid for 5 min and fifixed with 2% glutaraldehyde for 5 min, followed by washing with PBS for 3 times. And then, images of exosomes were captured with TEM (H-7700; Hitachi, Tokyo, Japan).

### Optimization of experimental conditions

We tried to determine the optimal stimulation concentration and time of TG stimulating PC3 cells. Four concentrations (0, 1, 2, 2.5, 3, and 4 µmol/l, respectively) of TG were added into PC3 cells in the normal condition for 24 h. After 24 h, the cells were collected, and proteins were extracted. The expression of C/EBP homologous protein (CHOP) and glucose‑regulated protein 78 (GRP78) of PC3 cells were determined by western‑blotting to determine the optimal stimulation concentration. After we chose the final stimulation concentration of 3 µmol/l TG, we set 0, 3, 6, 12, 24 and 48 h separately as the stimulation time points. Finally, we collected PC3 cells and extracted proteins. western‑blotting was used to detect the expression of the proteins CHOP and GRP78 at different time points to determine the optimal stimulation time.

We determined the optimal stimulation concentration of TG-stimulated PC3 cells-derived exosomes. Four concentrations (10, 20, 30, and 40 µg/ml, respectively) of exosomes were added to THP-1 macrophages induced by PMA in the normal condition for 24 h. The expression of PD-L1 of macrophages was determined by western‑blotting to determine the optimal stimulation concentration.

### Exosome labeling and tracing

Purified PC3-derived exosomes isolated from the culture medium were collected and labeled with PKH67 Green Fluores-cent membrane linker dye (Millipore, Sigma, Burlington, MA, USA) according to manufacturer's instructions. Then, the labeled exosome pellets were resuspended and added to the unstained macrophages for 24 h for exosomes internalization studies and the nuclei were stained with DAPI. Finally, the macrophages were examined and photographed with laser scanning confocal microscope (Carl Zeiss, Oberkochen, Germany).

### Western blotting

The total protein content from cells or exosomes was extracted, subjected to SDS-polyacrylamide gel electrophoresis and then transferred onto PVDF membranes. The PVDF membranes were blocked with 5% non-fat dry milk in PBS + 0.05% Tween-20 and then incubated with primary antibodies anti-GRP78 (1:2,000; Abcam), anti-CHOP (1:20,000; Abcam), anti-CD63 (1:2,000; Abcam), anti-Calnexin (1:2,000; Cell Signaling Technology), anti-TSG101 (1:50,000; Abcam), anti-PI3K/p-PI3K (1:1,000; Abcam), anti-AKT/p-AKT (1:1,000; Abcam) and GAPDH (1:35,000; Proteintech) overnight at 4 °C. Then they were washed with PBST for 3 times and incubated for 2 h at room temperature with the secondary antibodies conjugated to HRP. The bands were detected by using a gel imaging analysis system.

### qRT-PCR

The total RNA from CON-EXO group and TG-EXO group macrophages was isolated according to the manufacturer's instructions. The relative expression levels of IL-6, IL-10, TGF-β, IL-1β, IL-12 and TNF-α mRNA were calculated by the 2^-ΔΔCT^ and normalized to the expression of GAPDH. The expression levels of the mRNAs were reported as fold changes vs. control. RT-PCR primers were designed according to previous research 7-9.

### Cytometric beeds array (CBA)

CON-EXO and TG-EXO with a final concentration of 30 µg/ml were co-cultured with THP-1 macrophages induced by PMA for 24 hours. The supernatant was collected and the expressions of IL-6, IL-10, TGF-β, IL-1β, IL-12 and TNF-α cytokines were detected by human CBA cytokine kit according to the instructions.

### Flow cytometry

Macrophages of CON-EXO and TG-EXO group were washed with cold PBS and separated with 5 mM ethylene diamine tetraacetic acid. Then, the macrophages (0.5 × 10^6^) were resuspended in 1 mL PBSand stained by incubation with mAb: PerCP-Cy5.5-conjugated anti-human CD11c, APC-Cy7-conjugated anti-human CD206, PE-conjugated anti-human CD192, FITC-conjugated anti-human CD16, and APC-conjugated anti-human CD274 (PD-L1). Fifty thousand cells were measured per sample. Positive cell percentages were analyzed by flow cytometry (BD FACScan using Cell Quest Pro Software, BD Biosciences).

### Statistical analysis

Statistical analysis was performed by using SPSS 19.0 software (IBM SPSS, Chicago, USA). Differences between two groups were calculated, using ANOVA method, a P value < 0.05 was defined as statistically significant.

## Results

### Optimization of experiment conditions

**Figure 1:**
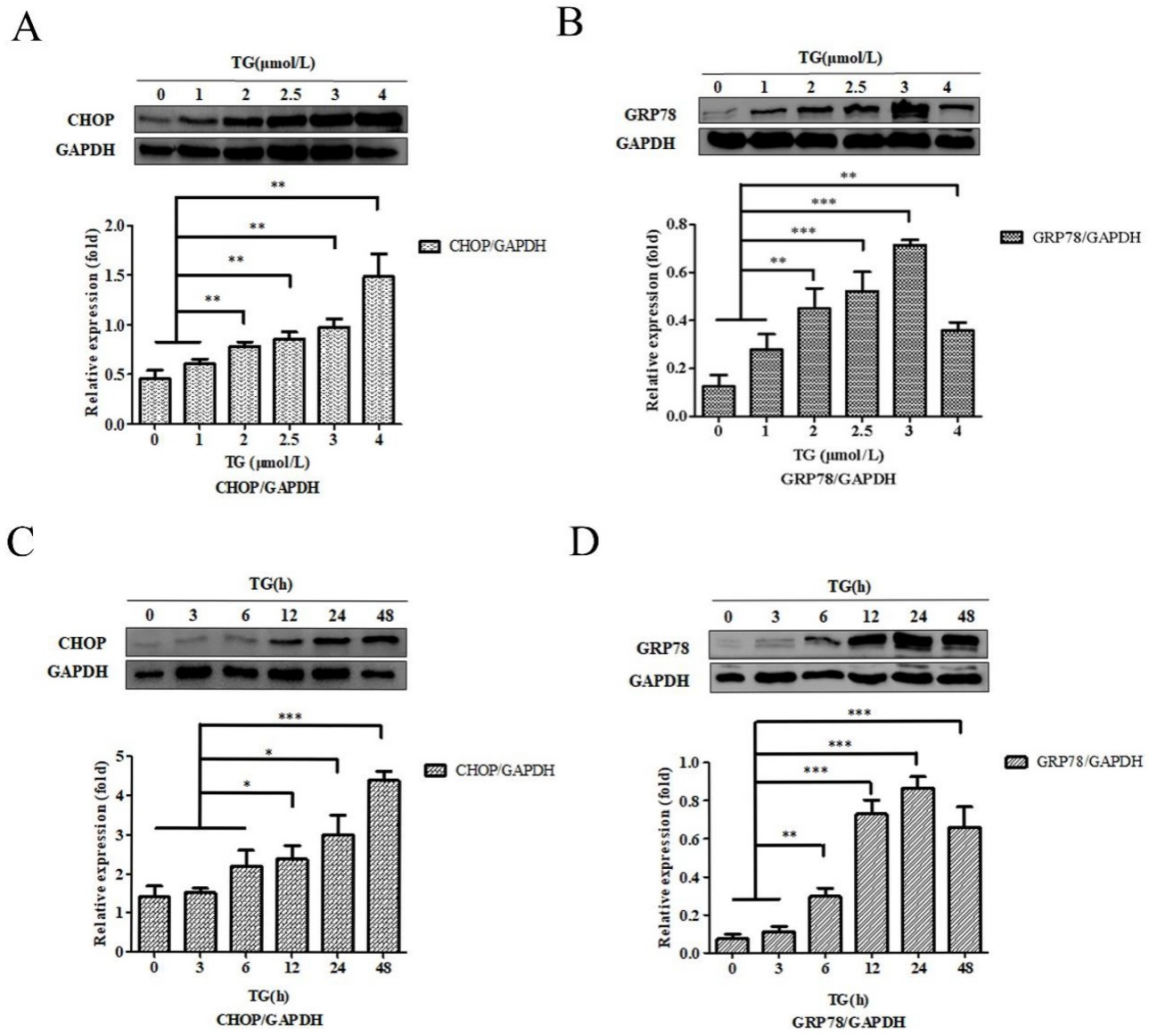
** Characteristics of ER stress‑associated PC-3 cells.** (A, B) PC-3 cells were treated with 0, 1, 2, 2.5, 3 and 4 µM TG for 24 h. The expressions of CHOP and GRP78 protein were analyzed, relative to GAPDH intensity. (C, D) PC-3 cells were treated with 3 µM TM for 0, 3, 6, 12, 24 and 48 h. The expressions of CHOP and GRP78 protein were measured, relative to GAPDH intensity. The results are expressed as mean ± SD of at least three independent experiments. *P< 0.05,**P< 0.01, ***P< 0.001, vs. negative control.

PC-3 cells were co‑cultured, using different concentrations of TG for 24 h, and the CHOP and GRP78 protein expressions were detected. The results showed that the expression of CHOP and GRP78 reached to the maximum when PC-3 cells were incubated with 4.0 µM and and 3.0 µM TG respectively (Fig. 1A and B). PC-3 cells were also treated with 3.0 µM TG for 0, 3, 6, 12, 24 and 48 h. CHOP and GRP78 concentrations peaked at 48 hours and 24 hours, respectively. Therefore, treating PC-3 cells with 3.0 µM. TG for 24 h was considered the optical experiment conditions to induce ER stress in subsequent experiments (Fig. 1C and D).

### Exosomes samples were examined by TEM and western blotting

**Figure 2:**
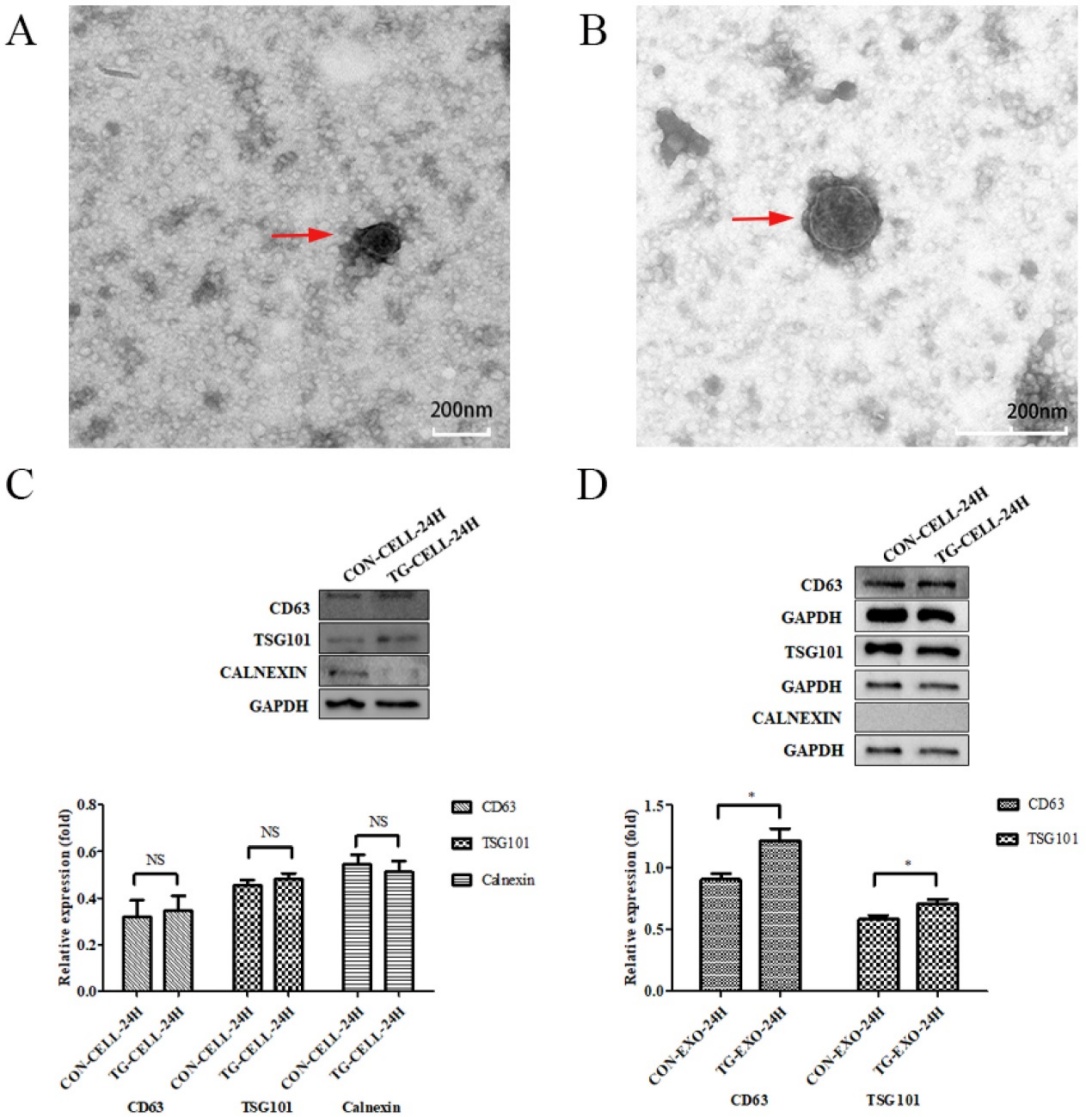
** Isolation and identification of exosomes.** (A, B) Exosomes isolated from the supernatants of PC-3 cells under TEM. (C, D) CD63, TSG101 and Calnexin expression in PC-3 cells and exosomes isolated from supernatant of samples were assessed by western blot, respectively.*P< 0.05. Representative images are shown.

TEM images of exosomes obtained from the supernatant of PC-3 cells under different conditions (untreated control, treated with TG) are shown in Figure 2A and Figure 2B, respectively. Exosomes were round or oval in shape, membrane vesicles with a size of 30-120 nm in diameter under TEM.

The results showed that exosomes were positive for the typical exosomal markers that are CD63 and TSG101, but negative for the endoplasmic reticulum marker, Calnexin. We confirmed that the isolated exosomes were free from cellular debris. The protein expression levels of CD63 and TSG101 in TG-EXO were higher than CON-EXO (Fig. 2C and Fig. 2D).

### Identification of THP-1 macrophages induced by PMA

**Figure 3:**
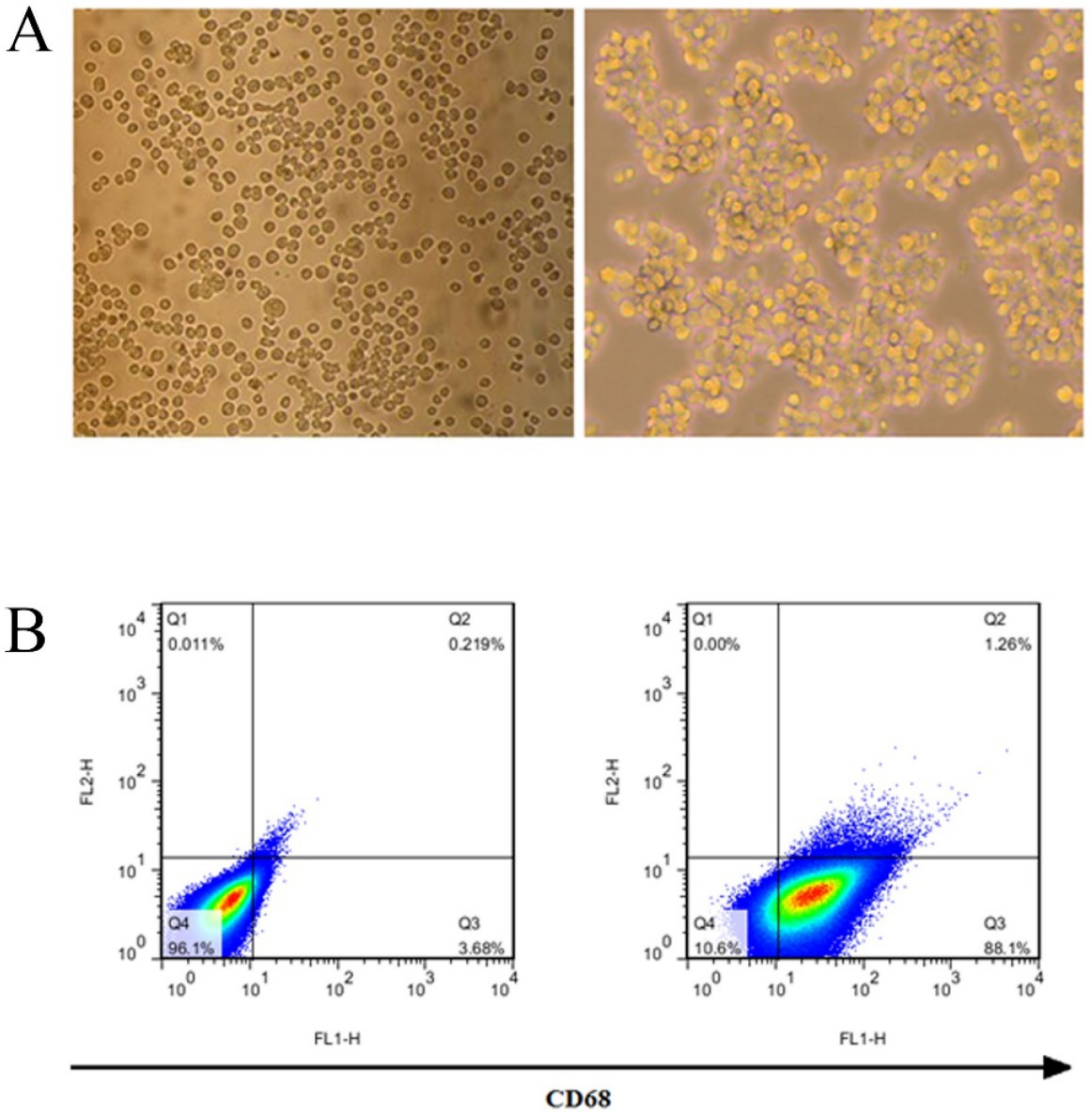
**Induction and identification of macrophages.** (A) THP-1 cells were transformed into macrophages by PMA. (B) The macrophage surface marker CD68 was detected by flow cytometry.

THP-1 was induced by 50 ng/ml PMA for 24 hours to become macrophages. THP-1 macrophages began to adhere to the wall after 6 hours, and their morphology changed, THP-1 cells are transformed into macrophages after being stimulated by PMA (Fig. 3A). The results of flow cytometry showed that THP-1 was induced to differentiate into macrophages, and its surface marker CD68 was highly expressed, with a positive rate of 89.36% (Fig. 3B).

### PC3-derived exosomes are internalized by macrophages

**Figure 4:**
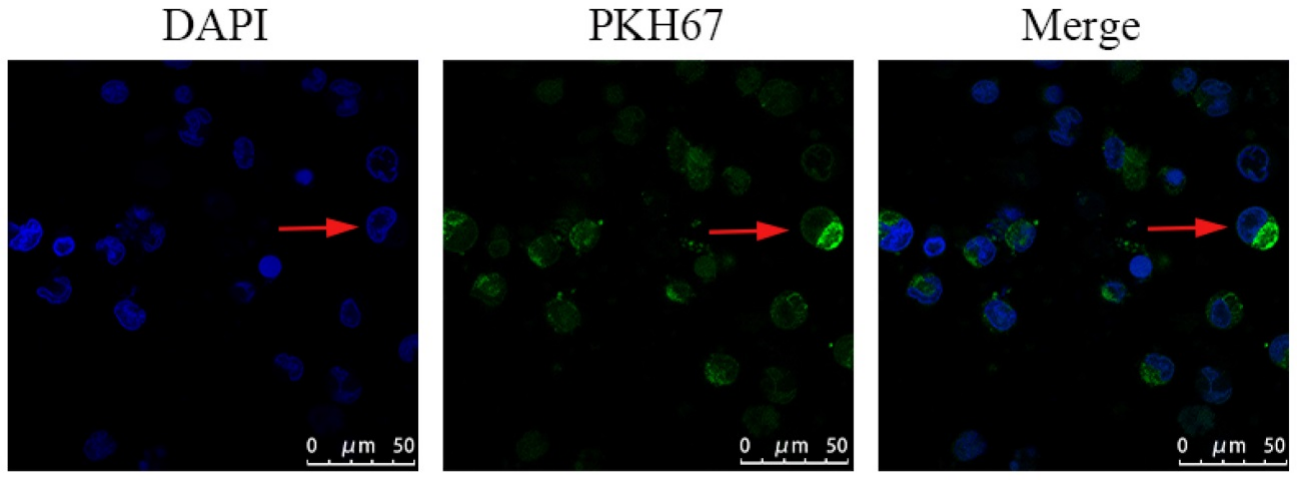
PKH67-labeled exosomes were co-incubated with mTHP-1 cells and examined by confocal microscopy. Confocal microscopy was used to measure the incorporation of PKH67‑labeled exosomes into RAW264.7 cells. Representative images are shown.

Confocal microscopy was used to measure the incorporation of PKH67‑labeled exosomes into macrophages. The PKH67 green fluorescent dye-labeled exosomes were localized in the cytoplasm of THP-1 macrophages, suggesting that PKH67-labeled PC3-derived exosomes could be effectively phagocytized by THP-1 macrophages (Fig. 4).

### Optimal concentration of PC-3 derived exosomes on macrophages

**Figure 5:**
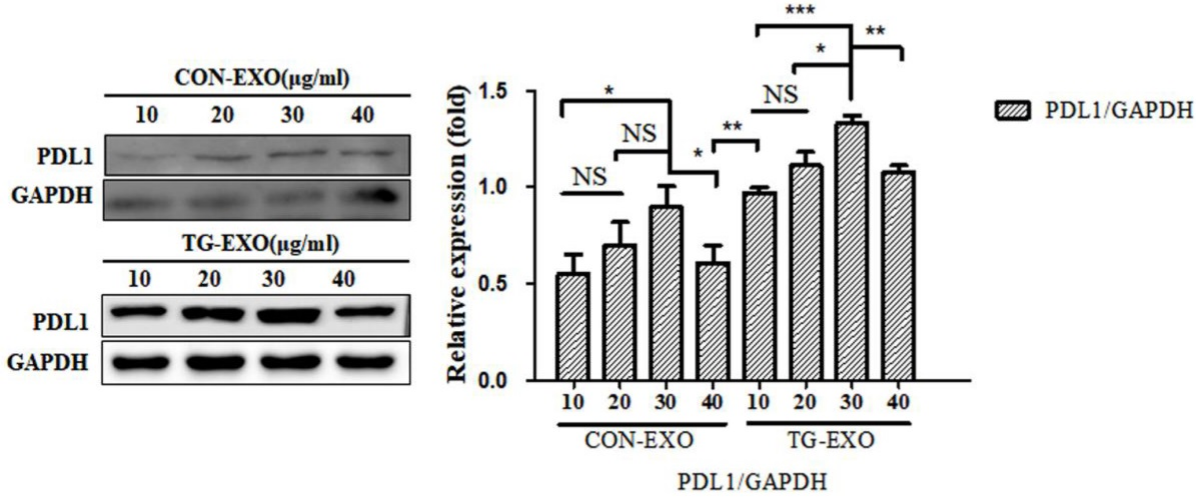
** TG-EXO increases macrophage PD-L1 expression.** The protein levels of PD-L1 were determined by western blot. Data are shown as the means ± SD of at least three independent experiments. *P< 0.05, **P< 0.01 with indicated groups.

TG-EXO and CON-EXO were added to macrophages to make their final concentrations 10 µg/ml, 20 µg/ml, 30 µg/ml and 40 µg/ml, and then continue to culture for 24 h. Western Blot was used to detect the expression level of PD-L1 protein in macrophages to determine the optimal concentration of exosomes acting on macrophages. Western Blot results showed that the final concentration of TG-EXO and CON-EXO in macrophages was 30 µg/ml, the protein concentration was the highest. This experimental condition is also used for subsequent experiments (Fig. 5).

### TG-EXO induces M2 polarization of macrophages

**Figure 6:**
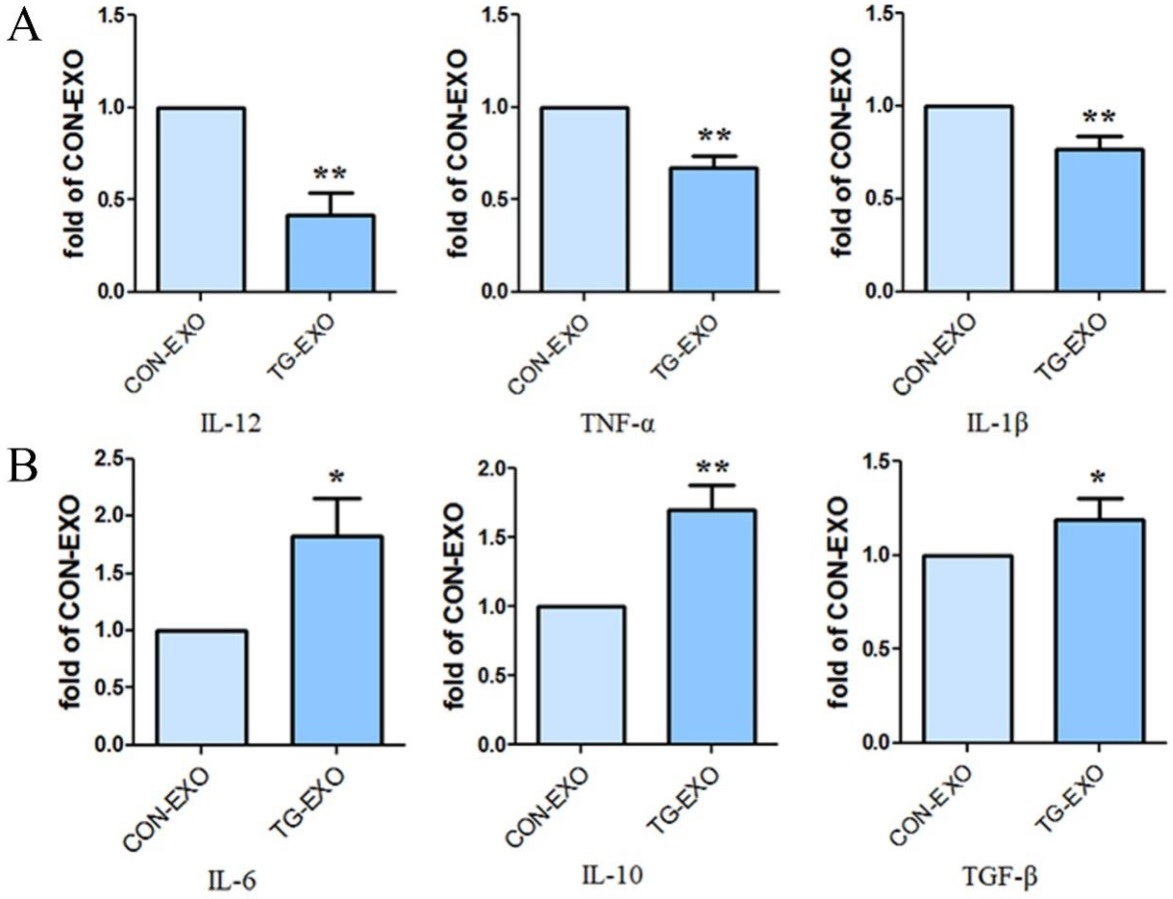
** TG-EXO promoted M2 polarization.** (A) IL-12, TNF-α and IL-1β (B) IL-6, IL-10 and TGF-β levels were measured using RT-PCR. *P< 0.05, **P< 0.01 with indicated groups.

Compared with CON-EXO group, the mRNA levels of macrophages associated inflammatory factors IL-12, TNF-α and IL-1β in TG-EXO group decreased significantly (Fig. 6A). Compared with CON-EXO group, macrophages released a large number of related inflammatory factors IL-6, IL-10 and TGF-β in TG-EXO group, and the difference was statistically significant (Fig. 6B).

### The expression level of TG-EXO and CON-EXO groups related inflammatory factors by CBA

**Figure 7:**
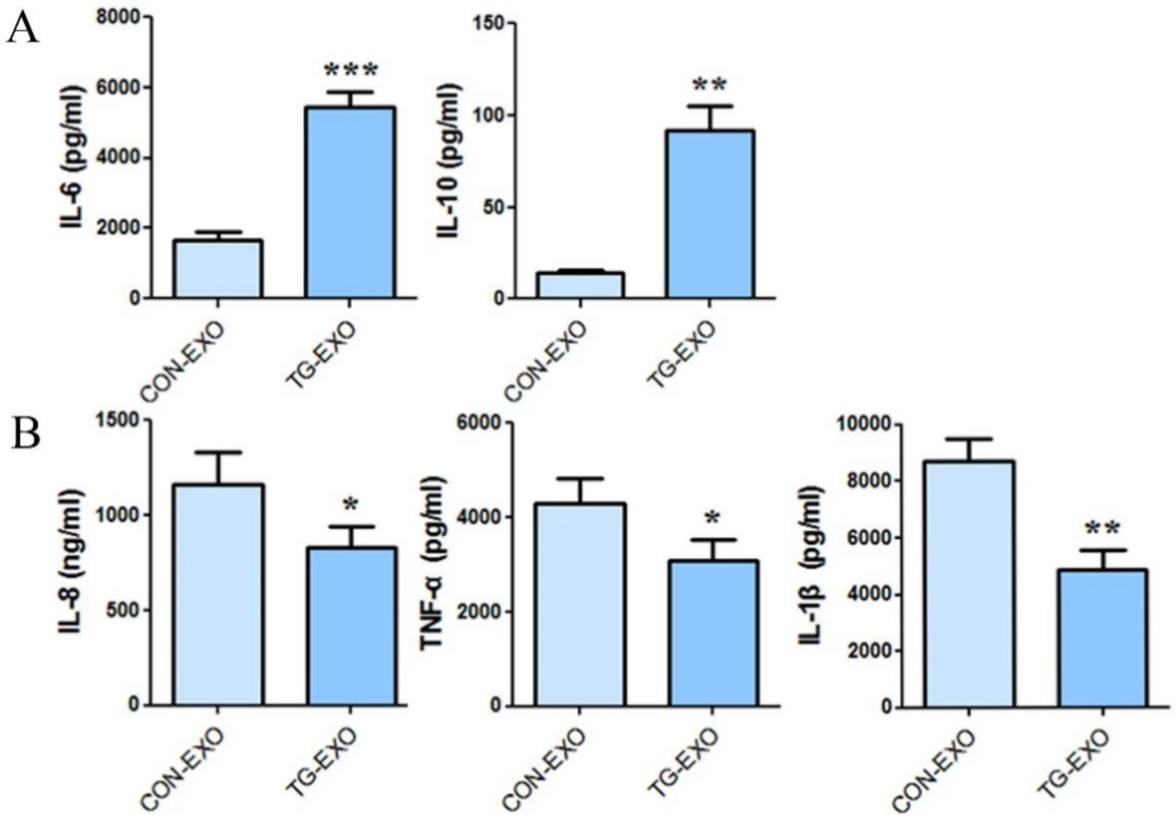
** TG-EXO promoted M2 polarization.** (A) IL-6 and IL-10, (B) IL-8, TNF-α and IL-1β levels were measured using a CBA inflammatory factor kit. *P< 0.05, **P< 0.01 with indicated groups.

CBA results showed that co-incubation of TG-EXO with THP-1 macrophages could increase the expression of IL-6 and IL-10 (Fig. 7A), and decreased the expression of IL-8, TNF-α and IL-1β compared with CON-EXO treated THP-1 macrophages (Fig. 7B).

### Detection of macrophage polarization induced by exosomes by flow cytometry

**Figure 8:**
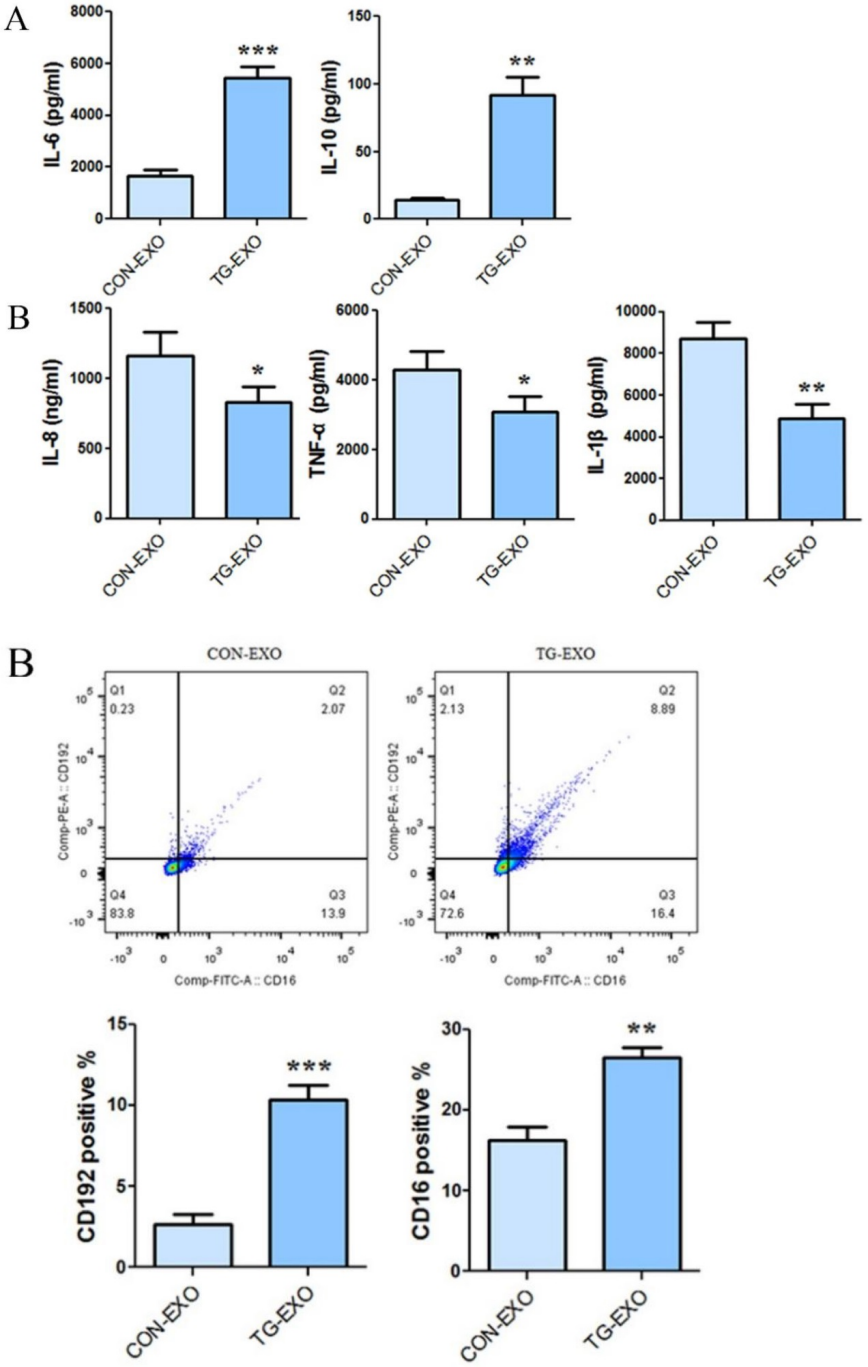

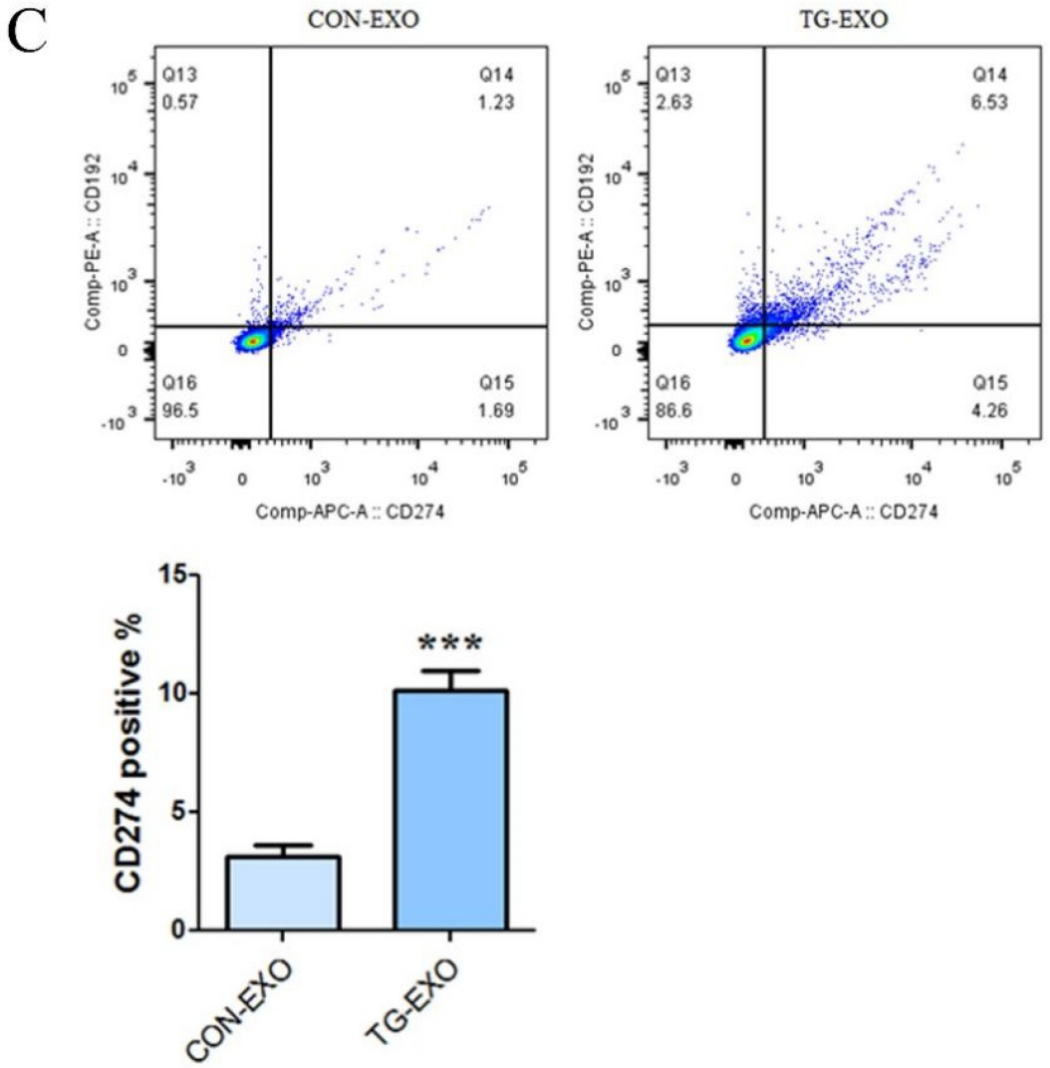
** Flow cytometry was used to detect the M2 markers.** CD206, CD16 and PD-L1 were used to check macrophages polarization under TG-EXO and CON-EXO. *P< 0.05, **P< 0.01 with indicated groups.

The levels of CD206, CD16 and PD-L1 on the surface of M2 macrophages were detected by flow cytometry. The results showed that compared with CON-EXO, TG-EXO could promote the polarization of THP-1 macrophages from M0 to M2, and the exosomes secreted by PC3 cells under endoplasmic reticulum stress could significantly promote the expression of M2 surface markers CD206 (Fig. 8A), CD16 (Fig. 8B) and PD-L1 (Fig. 8C) of THP-1 macrophages.

### TG-EXO can induce M2 polarization of macrophages by activating PI3K/Akt signaling pathway

**Figure 9:**
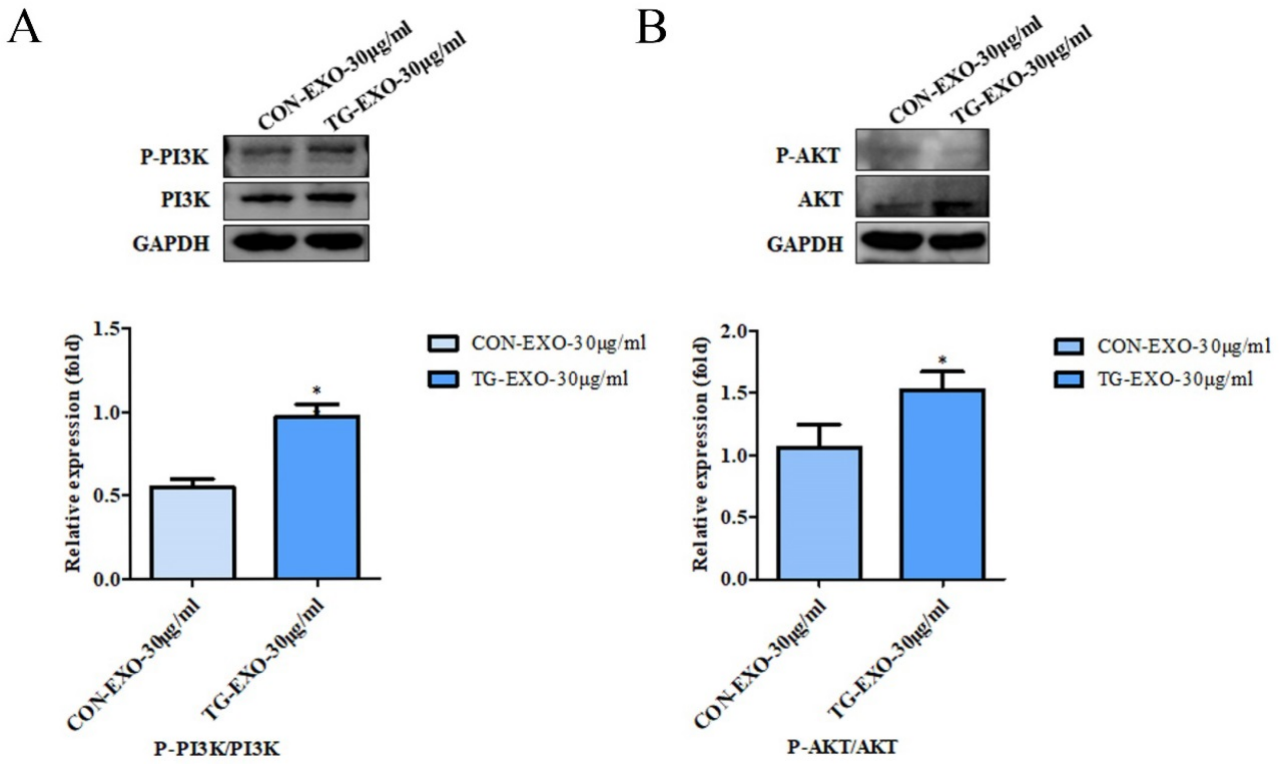
** TG-EXO enhances cytokine expression by activating the PI3K/Akt pathway.** Protein expression levels of the PI3K/AKT and P-AKT/AKT pathway were measured by western blot analysis. Blots presented in (A) P-PI3K/PI3K and (B) P-AKT/AKT, relative to GAPDH intensity. Data are presented as the means ± SD of at least three independent experiments. *P< 0.05, **P< 0.01 as indicated.

Macrophages were incubated with TG-EXO or CON-EXO, WB results showed P-PI3K/PI3K (Fig. 9A) and P-AKT/AKT (Fig. 9B) relative expression levels were more prominent increased. These data suggested that TG-EXO could activate PI3K/AKT signaling pathway in THP-1 macrophages.

## Discussion

Prostate cancer (PCa) is a malignant tumor which seriously threatens the health of men in the world. Therefore, it is urgent for clinical and basic research to explore the potential mechanism of PCa and find potential treatment methods.

Exosomes can deliver nucleic acids and proteins, and mediate the communication among the cells. Substances in vesicles can transfer and change the signal pathway of recipient cells 11. Alternatively, they may be internalized by endocytosis and/or phagocytosis, and may even fuse with the target cell membrane, transferring their contents into the cytoplasm. Therefore, exosomes derived from donor cells can change the physiological state of recipient cells 14. Transmission electron microscopy can be used to examine the presence of exosomes. After purification, the exosomes were observed under transmission electron microscope. In this study, exosomes were round or quasi-round vesicle-like structures with double membranes, with a diameter of about 30-120 nm. They were scattered and relatively uniform in size under transmission electron microscopy. In addition, Western blotting can be used to detect several secrete markers (such as CD9, CD63 and CD81) for the identification of secretions 15. In the experiment, we used ERS to marker CHOP and GRP78 as reference indicators, it was determined that the most appropriate concentration of TG for PC3 cells to cause ERS was 3 µmol/L, and the optimal time was 24 hours. The results of Western blotting showed that the exosomes secreted by PC3 cells had the expression of exosome marker CD63 and TSG101, but there was no expression of endoplasmic reticulum protein calnexin, which showed that the exosomes extracted in this experiment did not contain cell components and had high purity 18.

TME has attracted more and more attention because of its overall role in regulating tumor progression and metastasis. As the most abundant immune cell population in TME, macrophages are the most abundant inflammatory cells, also known as tumor-associated macrophages (TAMs), which may show different phenotypes in response to various stimuli in the local microenvironment. In general, macrophages can differentiate into proinflammatory classic activated M1 macrophages, which can secrete proinflammatory cytokines such as IL-1, IL-8, IL-12 and TNF-α, mediating inflammatory response and anti-tumor immunity. M1 macrophages are generally considered to be effector cells that kill microorganisms and tumor cells and produce a large number of pro-inflammatory cytokines. However, based on the characteristics of immunosuppression, M2 macrophages play the opposite role, clearing debris, promoting angiogenesis, tissue remodeling, repairing anti-inflammatory and promoting tumor effects, which are characterized by high IL-10 and low IL-12 phenotypes 19-21.

To determine the microenvironmental mechanisms underlying the formation of TAMs through exosome-mediated communication between tumor cells and immune cells, we used the human acute monocytic leukemia cell line (THP-1), which is the most commonly used to study monocyte/macrophage differentiation and function. We found that M0 macrophages were successfully obtained after THP-1 cells were stimulated by PMA for 24 hours. 22. Current studies have shown that exosomes can polarize M0 macrophages into M2 phenotype, as evidenced by specific cytokine production and phenotypic changes in surface markers. In this study, we used exosomes secreted by PC3 cells under ERS to stimulate macrophage polarization. In addition, the results showed the exosomes labeled with PKH67 green fluorescent dye could be effectively phagocytized by M0 macrophages under laser confocal microscope. Macrophages have high functional plasticity and heterogeneity, hypoxia, ischemia and low pH TME can cause endoplasmic reticulum stress in tumor cells, so as to enhance the immunosuppressive ability of TAMs and promote the infiltration and polarization of macrophages 24. The results of this study showed that TAM had the characteristics of M2 polarization, such as increased expression of inflammatory cytokine IL-10 and decreased expression of IL-12. IL-10 could secrete immunomodulatory anti-inflammatory factors that influence the activity of many cell types in the immune system, while IL-12, as a proinflammatory factor, plays a key role in Th1 cell development.

Transforming growth factor β (TGF-β) is a multifunctional growth factor produced by cancer cells, macrophages and fibroblasts, with a wide range of immunosuppressive effects promoting tumor and regulatory T cell function. In this study, the co-incubation of TG-EXO with macrophages polarized the macrophages towards M2, and the expression of TGF-β was significantly increased (P<0.05).

As an immunomodulator, IL-6 can stimulate tumor cell proliferation, reduce apoptosis, induce drug resistance, and promote bone metastasis. TNF-α and IL-1 secreted by macrophages are strong stimulators of IL-6 produced by tumor cells. Macrophages stimulate IL-6 produced by tumor cells and promote progressive growth of bone metastasis of prostate cancer through their positive feedback. This positive feedback can produce chemotaxis of macrophages, and then produce TNF-α, resulting in increased IL-6 produced by tumor cells 22. The results showed that TG-EXO were co-cultured with macrophages could promote macrophages to secrete IL-6 and induce them to transform into tumor-associated M2 macrophages.

RT-PCR was used to detect changes in the mRNA levels of macrophage polarization-related inflammatory factors induced by exosomes. Compared with CON-EXO, the expression of macrophage-associated inflammatory cytokines IL-12, TNF-α and IL-1β was significantly decreased in TG-EXO treatment (P< 0.05), and the expressions of IL-6, IL-10 and TGF-β was increased (P< 0.05). This result verifies the polarization of M0 macrophages to M2 after TG-EXO stimulated M0 macrophages in the transcriptional level. In addition, we detected the changes of macrophage-related inflammatory cytokines affected by exosomes by CBA. The results showed that compared with CON-EXO, the co-incubation of TG-EXO with macrophages significantly decreased the expression of macrophage-associated inflammatory cytokines IL-8, TNF-α and IL-1β, while increased the expression of IL-10 and IL-6 (P<0.05), which was consistent with the above results. These results suggested that TG-EXO can induce M2 polarization and regulate the expression of related inflammatory factors.

More and more studies have shown that the M2 polarization of macrophages is now considered to be related to the secretion of inflammatory factors and the expression of some specific markers of M2 macrophages such as the mannose receptor CD206 23. Programmed death ligand 1 (PD-L1) is a co-suppressor that is expressed in a variety of tumor cells and immune cells and plays an important role in blocking cancer immunity by binding programmed death receptor 1 (PD-1) 27. It has been found that tumor microenvironment-related factors can upregulate the expression of PD-L1 and provide inhibitory signals for T cell activation, so as to inhibit the anti-tumor immune response. These results will provide an insight into the mechanisms which cancer evades immune surveillance 29. In our study, CD16, CD206 and PD-L1 levels were detected by flow cytometry, respectively. The results showed that compared with CON-EXO, TG-EXO significantly increased the expression levels of M2 macrophages markers CD16, CD206 and PD-L1 (P < 0.01). Our study reveals a new mechanism of immune escape mediated by TAMs in the tumor microenvironment, which mediates immune escape by upregulating the expression of macrophage PD-L1.

Inflammatory signaling pathway is considered to be a key pathway of macrophage polarization 23. Phosphatidylinositol-3 kinase (PI3K) signaling pathway is one of the most famous cancer survival pathways and one of the most important alternative pathways for prostate cancer 30. PI3K is a plasma membrane-associated protein kinase. Once activated, it will catalyze the phosphorylation of PIP2 to produce PIP3. PIP3 then activated intracellular signals by binding to homologous domains of many signal proteins, including AKT. AKT is the most common active tyrosine kinase in metastatic prostate cancer 31-34. Activated AKT is a kinase, which is phosphorylated in turn and activates many oncogenic features in cancer cells. Once phosphorylated at Ser473 and Thr308, activated AKT can activate many downstream functions through its kinase activity 30. The PI3K/AKT signaling pathway regulated cell metabolism, tumorigenesis, growth, proliferation, metastasis and cytoskeleton reorganization. It played an important role in the proliferation and differentiation of cancer cells. In order to further explore whether TG-EXO can induce macrophages to M2 polarization by activating PI3K/AKT signaling pathway, the protein expression of PI3K/AKT in macrophages co-incubated with TG-EXO or CON-EXO was detected by Western blotting in this study. The results showed that compared with CON-EXO, the expression of PI3K/AKT protein increased in macrophages co-incubated with TG-EXO, p-PI3K/PI3K and p-AKT/AKT were expressed in THP-1 macrophages treated with TG-EXO were significantly higher than CON-EXO. This suggests that the essence of macrophage polarization induced by exosomes secreted by tumor cells under endoplasmic reticulum stress may regulate gene expression in macrophages by activating PI3K/Akt signaling pathway, but the more detailed process at the molecular level remains to be explored.

In conclusion, the co-culture of exosomes secreted by PC3 cells in ERS and macrophages induced by THP-1 leads to M2-type polarization of macrophages, mainly manifested that the high expression of PD-L1 and the release of a large number of inflammatory cytokines such as IL-6 and IL-10. These results indicated that prostate cancer cells in TME may transmit their stress signal to macrophages among exosomes through the PI3K/AKT signaling pathway, leading to phenotypic and functional changes of macrophages and promote the expression of immunosuppressive factors in TAM, so as to enable tumor cells to evade immune surveillance, spread and metastasize.

## Conclusion

In conclusion, our study proved that carotene (TG) can induce endoplasmic reticulum stress and secrete more exosomes in prostate cancer cells at a certain dose and time. The exosomes released by prostate cancer cells under endoplasmic reticulum stress can be effectively taken up by macrophages, and affect the polarization of macrophages to M2 by up-regulating the expression of PD-L1 and inflammatory factors. Exosomes act as a bridge to transmit information among cells, and there is a significant correlation between PI3K/Akt signaling pathway and the risk of prostate cancer.
